# Ability of visual assessment of left ventricular dyssynchrony by cine-MRI to identify potential responders to cardiac resynchronization therapy

**DOI:** 10.1186/1532-429X-11-S1-P264

**Published:** 2009-01-28

**Authors:** Kai Muellerleile, Loant Baholli, Michael Groth, Katharina Koopmann, Achim Barmeyer, Ralf Koester, Gerhard Adam, Thomas Meinertz, Stephan Willems, Gunnar Lund

**Affiliations:** 1 University Medical Center Hamburg-Eppendorf, Hamburg, Germany; 2 Center for Cardiology and Cardiovascular Surgery, Hamburg, Germany

**Keywords:** Public Health, Ejection Fraction, Left Ventricular, Strong Correlation, Cardiac Resynchronization Therapy

## Objective

To assess the ability of cine-MRI to identify potential responders to cardiac resynchronization therapy (CRT).

## Methods

40 patients with reduced ejection fraction (28 ± 11%), with (n = 21) or without (n = 18) left bundle branch block underwent cine-MRI and tissue-doppler imaging echocardiography (TDI). Time to peak systolic velocity (TPV) was measured by TDI for the septal, anterior, lateral and inferior left ventricle (LV). Time to peak contraction (TPC) was assessed visually for corresponding regions by cine-MRI. Left ventricular (LV) dyssynchrony was defined as the maximum delay of TPV- and TPC between opposing regions for TDI and cine-MRI, respectively. Correlation and agreement between TDI and cine-MRI were calculated. The ability of cine-MRI to identify patients with relevant echocardiographic LV dyssynchrony (defined as >65 ms) was assessed by ROC analysis.

## Results

A strong correlation between LV dyssynchrony by TDI and cine-MRI was found (r = 0.84, P < 0.0001). Bland-Altman analysis showed a large difference of absolute values between both methods (mean difference 108 ± 104 ms). ROC analysis revealed an optimal cut-off value for cine-MRI of > 145 ms to identify patients with relevant echocardiographic LV dyssynchrony (sensitivity 89%, specificity 95%). The ability of cine-MRI to discriminate between patients with and without relevant echocardiographic LV dyssynchrony was excellent (area under the curve 0.98; P < 0.0001).

## Conclusion

Visual assessment of LV dyssynchrony by cine-MRI can identify potential responders to CRT.

